# Validation of Ion Torrent^TM^ Inherited Disease Panel with the PGM^TM^ Sequencing Platform for Rapid and Comprehensive Mutation Detection

**DOI:** 10.3390/genes9050267

**Published:** 2018-05-22

**Authors:** Abeer E. Mustafa, Tariq Faquih, Batoul Baz, Rana Kattan, Abdulelah Al-Issa, Asma I. Tahir, Faiqa Imtiaz, Khushnooda Ramzan, Moeenaldeen Al-Sayed, Mohammed Alowain, Zuhair Al-Hassnan, Hamad Al-Zaidan, Mohamed Abouelhoda, Bashayer R. Al-Mubarak, Nada A. Al Tassan

**Affiliations:** 1 Behavioral Genetics Unit, Department of Genetics, King Faisal Specialist Hospital & Research Center, P.O. Box 3354, Riyadh 11211, Saudi Arabia; almostafaa@sdl.com.sa (A.E.M.); bbaz@kfshrc.edu.sa (B.B.); bashairrashid@hotmail.com (A.I.T.); naltassan@kfshrc.edu.sa (N.A.A.T.); 2 Department of Genetics, King Faisal Specialist Hospital & Research Centre. P.O. Box 3354, Riyadh 11211, Saudi Arabia; tfaquih@kfshrc.edu.sa (T.F.); fahmad@kfshrc.edu.sa (F.I.); kramzan@kfshrc.edu.sa (K.R.); mabouelhoda@yahoo.com (M.A.); 3 Department of Medical Genetics, King Faisal Specialist Hospital & Research Centre, P.O. Box 3354, Riyadh 11211, Saudi Arabia; moeen@kfshrc.edu.sa (M.A.-S.); alowain@kfshrc.edu.sa (M.A.); zhassnan@kfshrc.edu.sa (Z.A.-H.); hzaidan@kfshrc.edu.sa (H.A.-Z.); 4 Saudi Human Genome Program, King Abdulaziz City for Science & Technology, Riyadh, Saudi Arabia; ra.kattan@gmail.com (R.K.); abdulbenissa@gmail.com (A.A.-I.)

**Keywords:** inherited diseases, mutations, gene panel, targeted NGS, AmpliSeq Inherited Disease Panel, Saudi Human Genome Program database

## Abstract

Quick and accurate molecular testing is necessary for the better management of many inherited diseases. Recent technological advances in various next generation sequencing (NGS) platforms, such as target panel-based sequencing, has enabled comprehensive, quick, and precise interrogation of many genetic variations. As a result, these technologies have become a valuable tool for gene discovery and for clinical diagnostics. The AmpliSeq Inherited Disease Panel (IDP) consists of 328 genes underlying more than 700 inherited diseases. Here, we aimed to assess the performance of the IDP as a sensitive and rapid comprehensive gene panel testing. A total of 88 patients with inherited diseases and causal mutations that were previously identified by Sanger sequencing were randomly selected for assessing the performance of the IDP. The IDP successfully detected 93.1% of the mutations in our validation cohort, achieving high overall gene coverage (98%). The sensitivity for detecting single nucleotide variants (SNVs) and short Indels was 97.3% and 69.2%, respectively. IDP, when coupled with Ion Torrent Personal Genome Machine (PGM), delivers comprehensive and rapid sequencing for genes that are responsible for various inherited diseases. Our validation results suggest the suitability of this panel for use as a first-line screening test after applying the necessary clinical validation.

## 1. Introduction

The morbidity, mortality, and disability that are associated with inherited diseases can be greatly reduced or prevented through improving the accuracy and speed of molecular testing. Genetic advances over the past decades led to the development of different mutation screening techniques, which have been applied in research or integrated into diagnostic laboratory protocols [1,2,3,4,5]. Sanger sequencing, for instance, is widely used for the genetic evaluation of inherited diseases, owing to its ability to detect point mutations and small insertion/deletion sequence changes with high accuracy. However, this method is typically utilized to evaluate a small fragment of a single gene, and in some (less common) cases, to test a limited number of genes simultaneously. Even with the wide availability of this method, it can be very expensive, labor intensive, and time consuming when being used to interrogate diseases with genetic diversity [6,7]. This is often the case with inherited diseases, in which various genes can be involved in the development of a monogenic disorder. The fast development and the increasing availability of several next generation sequencing (NGS) technologies, namely, whole genome, whole exome, and target panel-based sequencing allowed for comprehensive, high resolution, and accurate interrogation of the genome or portions of it in a short time. These features made NGS a valuable tool for discovery research and for clinical genetic diagnostics [8,9]. 

Targeted NGS is becoming the preferred method for identifying causal mutations in genetically heterogeneous disorders, such as cardiomyopathies, retinopathies, hearing loss, epilepsies, immunodeficiency, and ataxias [10,11,12,13,14,15,16]. A wide range of panels comprising either preselected or customized assortment of genes associated with a single or a group of conditions are commercially available (either as a service or ready-to-use) and are widely used to detect germline or somatic mutations [17,18,19,20]. Ion Torrent sequencing platform has been shown to perform well in terms of run time and total cost [21]. These are two important considerations when developing assays for use in routine clinical diagnostics. In 2012, Ion Torrent released its first version of the comprehensive panel for hereditary diseases, known as AmpliSeq Inherited Disease Panel (IDP, http://www.edgebio.com/sites/default/files/IonAmpliSeq_InheritedDiseasePanel_Flyer_CO25400_May%2023%202012.pdf). The recent version consists of 328 genes underlying over 700 inherited diseases. Therefore, we aimed here to assess the performance of the IDP as a sensitive and rapid comprehensive genetic assay. We randomly selected patients with inherited diseases in whom causal mutations were previously identified via Sanger sequencing for validating the detection yield of IDP.

## 2. Materials and Methods

### 2.1. Samples

**Figure 1:**
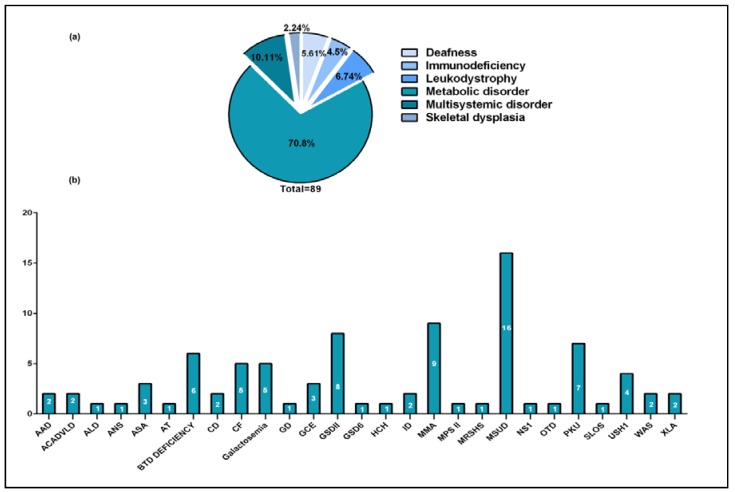
Disease categories and number of samples tested for each condition. (**a)** Samples breakdown by disease category. (**b)** Number of samples studied for each disease.

We randomly selected samples from 88 Saudi patients with previously confirmed genetic diagnosis after obtaining full informed consent from all of the participants. Written informed consents were obtained from all of the study subjects, which is in adherence with the declaration of Helsinki, and according to King Faisal Specialist Hospital & Research Centre (KFSHRC) Institutional Review Board (IRB) and Research Advisory Committee (RAC) rules and regulations under the following approved projects: (RAC#2020011, approved-present), (RAC#2050022, approved-present) (RAC#2100001, approved-present). The conditions and corresponding genes that were tested in this study are listed in Table 1. A breakdown of our validation cohort by disease category and the number of samples tested for each condition is summarized in Figure 1. DNA was extracted from whole blood using the QIAamp DNA Blood Mini Kit (Qiagen, Hilden, Germany). The quantity of extracted DNA was estimated using the broad range Qubit^®^ 2.0 kit (Invitrogen, Carlsbad, CA, USA), according to manufacturer’s instructions.

### 2.2. Library Building and Sequencing

Ten nanograms of DNA were used in three primer pools, in conjunction with Ion AmpliSeq Library Kit 2.0 (Thermo Fisher, Carlsbad, CA, USA) for 14 cycles. Pooled PCR amplicons were first digested using FuPa reagent (Thermo Fisher), and then ligated with universal adaptors. A maximum of two samples were pooled for emulsion PCR (ePCR) using the Ion OneTouch™ 200 Template Kit (Thermo Fisher). The ePCR template Ion Sphere particles were enriched using the Ion OneTouch ES (Thermo Fisher), following the manufacturer’s instructions. To assess the enrichment status, we used Ion Sphere Quality Control Kit (Thermo Fisher), and the products were measured using Qubit^®^ 2.0 Fluorometer (Thermo Fisher) following manufacturer’s instructions. Template positive Ion Sphere particles were sequenced using the Ion PGM™ 200 Sequencing Kit (Thermo Fisher) on the Ion Personal Genome Machine (PGM) and 318^TM^ semiconductor chip. 

### 2.3. Data Analysis

The bed files containing targeted regions corresponding to the IDP panel were generated based on human genome 19 (hg19) build and used for sequence reads alignment and analysis. Resulting aligned reads (BAM files) were visualized and interrogated with Integrative Genomics Viewer software (http://www.broadinstitute.org/igv). To generate good quality reads, adaptor sequences were trimmed and low quality reads were excluded before using Torrent Suite v4.0 with Variant Calling plugin for the initial analysis (Thermo Fisher, https://github.com/iontorrent/TS). The obtained variants were then annotated using public and in-house databases, as previously described [22]. The Variant calling plugin was set to include variants with a minimum coverage of 20×, following the software developers’ recommendations. On average, each variant calling file (VCF) contained around 800–1200 variants/sample, which was reduced after initial filtration to an average of 20 variants/sample.

### 2.4. Tertiary Analysis and Variant Validation

Tertiary analysis was carried out, as previously described [22,23], after applying more stringent quality filtering criteria of a minimum coverage of 50× and a quality score of at least 350 for single nucleotide variants (SNVs) and 700 for Indels. Briefly, after passing the initial quality check, the VCFs underwent a step-wise filtering process by two independent trained researchers that were both blind to the phenotype and the original mutation of each sample. The first step of the filtering process involved excluding intronic variants, synonymous variants, or variants present in international and/or local databases (Saudi Human Genome Program database-SHGP) (with MAF > 1%). In the second step, variants’ deleteriousness was assessed using three different prediction software (SIFT [24], PolyPhen-2 [25] and Mutation Taster [26]). Those predicted as “tolerated”, “neutral”, or “benign” were removed. In most cases only homozygous changes were selected, yielding a maximum of three variants/sample. Finally, the causative pathogenic variant was selected from the shortlist of variants, according to the clinical diagnosis. The final results were compared to the initial Sanger sequencing data to estimate the concordance. 

## 3. Results

### 3.1. Sequencing Quality, Coverage and Overall Panel Performance 

Sequencing and read mapping quality of the runs are summarized in Table S1. The overall average number of reads at Q17 was about 3.06 million per run, with an average base yield of about 392 Mbp. The panel generates a total of 10,309 amplicons with an average read length of 135 bp at Q0 and 118 bp at Q20. On average, 95% of these reads were aligned to the target regions (the target set of genes). The overall average depth was 191×. Target base coverage at 1×, 20×, and 50× are 98.36%, 93.29%, and 89.34%, respectively (Table S1). Also, 95% of the amplicons were free of strand bias. The average coverage per gene was ~98 (Table S2). Of the total amplicons, 253 (2.5%) had coverage less than 90%, and 105 (1%), from 78 genes, had suboptimal performance (Table S3). Overall, this shows good sequencing parameters for the runs that were included in the analysis. 

### 3.2. Variant Calling

For the 88 samples that were analyzed, an average of 1058 variants per sample were called and annotated. Recalling that the total length of the target region is 1,509,563, this translates to seven variants per 10,000 bases per sample. For the combined set of samples, the total number of unique variants was 11,118, with a rate of 7.36 variants per 1000 bases. 

### 3.3. Variant Detection Yield 

Validation yield was determined by evaluating the concordance between the original mutation and the NGS results for the corresponding case. However, two of the samples were excluded from the analysis due to either amplicon failure or design. Using our analysis pipeline, the IDP successfully detected 93.1% (97.3% for SNVs and 69.2% for Indels) of the mutations in our validation cohort (Table 2). Of note, the detection rate when including the two failed samples is 91%, (96% for SNVs and 64.3% for Indels). In addition, mutations that were masked in Table 2 were revealed to the researchers for the purpose of the analysis. Moreover, missing variants were attributed to low coverage, or the homopolymer effect (Table S4). 

## 4. Discussion

In this study, we report the successful validation of IDP as a comprehensive and sensitive assay for detecting causal mutations in a variety of inherited diseases. Using IDP with PGM, we achieved (~98%) sequence coverage of the targeted regions, with an average depth of 191X. A total of (1058) variants were detected in each sample before filtration. We were able to detect (93.1%) of the originally reported causal mutations. The remaining (6.9%) of causal variants were not detected due to the inadequate coverage of challenging DNA regions with homology or high GC-content (Table S4). 

A wide collection of disease-focused or comprehensive gene panels for inherited diseases is commercially available and is being used in clinical laboratories with various NGS platforms [17,27]. Examples of comprehensive inherited disease panels, other than the one assessed here, include Otogenetics and TruSight Inherited Disease Panel (Table S5). The panel from Otogenetics comprises the largest number of genes (~4500), however, the subsequent data analysis and the interpretation could be very challenging and time consuming. Besides, this panel is available only as a service. On the other hand, both the IDP and TruSight are available as predesigned ready-to-use panels. The TruSight covers 552 genes focused only on severe recessive child-onset diseases, whereas the IDP surveys 328 genes that are implicated in > 700 child or adult-onset inherited diseases. Both IDP and TruSight offer fast time-to-results. Delivering accurate results in a short turn-around time is imperative for any diagnostic test, as results may impact the treatment decision or prevent unnecessary interventions. 

When it comes to choosing the most appropriate genetic testing strategy, clinicians often face the challenge of deciding which NGS-based approach (targeted vs, Whole Exome Sequencing, WES) to pursue as first-tier genetic screening. Exome sequencing utilization in clinical settings (clinical exome sequencing) allows for the unbiased evaluation of roughly all 21,000 genes. This is crucial in situations of diagnostic uncertainty, in diseases with significant genetic and phenotypic heterogeneity or even to minimize the effect of diagnostic error as testing is not restricted to genes implicated in a certain disorder. One important advantage of exome sequencing is the capacity to identify alterations in both well characterized and novel genes, allowing for data re-analysis in the light of new gene-disease associations. Another advantage is that it can improve the management of patients by alerting physicians to unanticipated comorbidities that may alter the course of treatment or impact prognosis. On the other hand, technical limitations of this approach include incomplete gene coverage (especially in problematic regions), variant validation, and interpretation [28,29]. Another limitation to this approach is that it generates a long list of variants most of which are variants of uncertain significance (VUS) that are usually overlooked or are filtered out. Some of these variants could be clinically relevant (may represent actual mutations), however, unfeasible to validate. An additional ethical issue is, the disclosure of incidental/secondary findings, which are not uncommon [30,31].

Targeted gene panels have the potential to overcome some of the current exome sequencing limitations. They offer a superior coverage of up to 100% when coupled with Sanger sequencing [32]. Unlike exome sequencing, gene panel analysis generates substantially less variants, thus making the validation and interpretation much more efficient. This can minimize the chances of missing VUS with potential clinical relevance. More importantly, because the analysis is restricted to genes that are related to the primary clinical condition, the issue of incidental findings is reduced to a minimal concern in targeted-panels [27,33]. However, the major limitation of targeted-gene panels is that a panel could become obsolete if its content is not constantly updated to catch up with the fast pace of new genes discovery. 

With regard to the choice of NGS-based genetic testing, there seems to be a general consensus on using targeted-panels as first-tier genetic testing, particularly for diseases with distinct phenotypes and a good knowledge of the underlying genes. On the other hand, exome or genome sequencing are recommended to be reserved for those cases in which molecular diagnosis could not be established via targeted-panel testing [34,35]. Recently, a targeted sequencing approach using 13 different gene panels covering the majority of OMIM reported genes, demonstrated a high degree of clinical sensitivity and specify providing evidence for the advantages of utilizing targeted-panels over exome sequencing as first-tier genetic testing approach [22].

Due to the random selection of samples, the performance of the IDP could not be evaluated for all disease categories and certainly not all phenotypes. Our samples represented various disease categories with the majority being classified as metabolic disorders (Figure 1a). However, it is important to note that half of the Mendelian conditions that are prevalent in Saudi Arabia (Thalassemia, lysosomal storage disorders, hearing loss, organic acidemias, and retinal dystrophies) are covered by the IDP [36]. 

The assay in its current format is not intended to identify copy number variants, however, it is possible to incorporate algorithms for assessing this type of alteration into the bioinformatics pipeline after preforming the necessary validation [37]. Sensitivity validation results for this panel met the high degree that is required for research use. However, additional important quality measures, such as (run-to-run or laboratory-to-laboratory) reproducibility, should be evaluated before clinical implementation of the assay [38]. 

## 5. Conclusions 

This study demonstrated the suitability of the IDP as a rapid and comprehensive approach for screening a large number of genes that are responsible for over 700 different inherited diseases. It is worth mentioning that reported detection yield of gene panels for inherited diseases varies widely (24–95%), placing the rate achieved in our study at the upper range [22,23,39,40,41,42] 

Inherited diseases are expected to be frequently encountered in consanguineous populations. For instance, in Saudi Arabia, inherited conditions, such as Thalassemia, lysosomal storage disorders, hearing loss, organic acidemias, and retinal dystrophies are common [36,43]. In response to that, the Ministry of Health established two national molecular screening programs; newborn and premarital [44,45]. The incorporation of a comprehensive gene panel (such as the IDP and other available panels [22]) as a second-tier testing approach into any ongoing public screening programs would enhance their performance by improving diagnostic accuracy and expanding the range of conditions for which screening is available. 

## Figures and Tables

**Table 1:** List of inherited conditions tested in this study.

Clinical Diagnosis	Abbreviation	Category	Genes Included in the Panel	OMIM#
Agammaglobulinemia, X-linked, Type I	XLA	Immunodeficiency	*BTK*	300755
Argininosuccinate Lyase Deficiency	ASA	Metabolic disorder	*ASL*	207900
Arylsulfatase A Deficiency	AAD	Leukodystrophy	*ARSA*	250100
Ataxia Neuropathy Spectrum/Alpers Syndrome	ANS	Multisystemic disorder	*POLG*	203700
Ataxia-telangiectasia	AT	Multisystemic disorder	*ATM*	208900
Biotinidase Deficiency	BTD deficiency	Metabolic disorder	*BTD*	253260
Canavan Disease	CD	Leukodystrophy	*ASPA*	271900
Cystic Fibrosis	CF	Multisystemic disorder	*CFTR*	219700
Galactosemia	Galactosemia	Metabolic disorder	*GALT*	230400
Gaucher Disease	GD	Multisystemic disorder	*GBA*	230800
Glycine Encephalopathy	GCE	Metabolic disorder	*GCSH*, *GLDC*, *AMT*	605899
Glycogen Storage Disease Type VI	GSD6	Metabolic disorder	*GBE1*	232700
Hunter Syndrome/Mucopolysaccharidosis, Type II (MPS II)	MPS II	Metabolic disorder	*IDS*	309900
Hypochondroplasia	HCH	Skeletal dysplasia	*FGFR3*	146000
Inherited Deafness	ID	Deafness	*GJB2*, *GJB3*, *GJB6*, *COL11A2*, *KCNQ4*	220290
Maple syrup Urine Disease	MSUD	Metabolic disorder	*BCKDHA*, *BCKDHB*, *DBT*, *DLD*	248600
Marshall syndrome	MRSHS	Skeletal dysplasia	*COL11A1*	154780
Methylmalonic Acidemia	MMA	Metabolic disorder	*MMAA*, *MMAB*, *MMACHC*, *MUT*	251100
Noonan Syndrome (Types 1, 3, 4, 5 ,6)	NS	Multisystemic disorder	*KRAS*, *NRAS*, *PTPN11*, *RAF1*, *SOS1*	163950/609942/610733/611533/613224
Ornithine Transcarbamylase Deficiency	OTD	Metabolic disorder	*OTC*	311250
Phenylketonuria	PKU	Metabolic disorder	*PAH*	261600
Pompe Disease/Glycogen Storage Disease II (GSD II)	GSDII	Metabolic disorder	*GAA*	232300
Smith-Lemli-Optiz Syndrome	SLOS	Metabolic disorder	*DHCR7*	270400
Usher Syndrome Type 1	USH1	Deafness	*PCDH15*, *USH1C*, *CDH23*, *MYO7A*	276900/601067
Very Long Chain Acyl-Coenzyme A Dehydrogenase Deficiency	ACADVLD	Metabolic disorder	*ACADVL*	201475
Wiskott-Aldrich Syndrome	WAS	Immunodeficiency	*WAS*	301000
X-Linked Adrenoleukodystrophy	ALD	Leukodystrophy	*ABCD1*	300100

**Table 2:** List of mutations detected by AmpliSeq Inherited Disease Panel (IDP).

			Original Mutation		IDP Panel	
Case ID	Gene	Transcript ID	cDNA	Protein	Original Mutation Found (Y/N)	Genotype Matching (Y/N)
13-0045	*ABCD1*	NM_000033	c.1581C>A	p.Y527X	Y	Y
13-0051	*ACADVL*	NM_000018	c.65C>A	p.S22X	Y	Y
14-3258	*ACADVL*	NM_000018	**c.65C>A**	**p.S22X**	Y	Y
13-0097	*AMT*	NM_001164710	c.533G>A	p.R178H	Y	Y
13-0098	*AMT*	NM_001164710	c.280C>T	p.R94W	Y	Y
13-0059	*ARSA*	NM_000487	c.1055A>G	p.N352S	Y	Y
13-0095	*ARSA*	NM_000487	^#^Missense		Y	Y
13-0040	*ASL*	NM_001024943	c.1000C>T	p.Q334X	Y	Y
13-0131	*ASL*	NM_001024943	c.556C>T	p.R186W	Y	Y
13-0132	*ASL*	NM_001024943	c.544C>T	p.R182X	Y	Y
13-0133	*ASPA*	NM_000049	^#^Frameshift insertion		N	NA
14-3064	*ASPA*	NM_000049	^#^Frameshift deletion		N	NA
13-0020	*ATM*	NM_000051	c.381_381delA	p.V128*	Y	Y
13-0009	*BCKDHA*	NM_000709	c.905A>C	p.D302A	Y	Y
13-0013	*BCKDHA*	NM_000709	**c.905A>C**	**p.D302A**	Y	Y
13-0093	*BCKDHA*	NM_000709	c.1270C>T	p.Q424X	Y	Y
13-0094	*BCKDHA*	NM_000709	c.647-1G>C	NA	Y	Y
13-0096	*BCKDHA*	NM_000709	c.347A>G	p.D116G	Y	Y
13-0120	*BCKDHA*	NM_000709	c.808G>A	p.A270T	Y	Y
13-0135	*BCKDHA*	NM_000709	c.659_662delCGTA	p.Y221Qfs*108	Y	Y
13-0091	*BCKDHB*	NM_000056	c.286_288delGAA	p.E96del	N	NA
13-0092	*BCKDHB*	NM_000056	c.1A>T	p.M1L	N	NA
13-0129	*BCKDHB*	NM_000056	c.1006G>A	p.G336S	Y	Y
13-0053	*BTD*	NM_000060	^#^Missense		Y	Y
13-0061	*BTD*	NM_000060	^#^Frameshift deletion		Y	Y
13-0062	*BTD*	NM_000060	^#^Missense		Y	Y
13-0087	*BTD*	NM_000060	^#^Missense		Y	Y
13-0088	*BTD*	NM_000060	^#^Frameshift deletion		Y	Y
13-0019	*BTK*	NM_000061	c.763C>T	p.R255X	Y	Y
13-0028	*BTK*	NM_000061	c.982C>T	p.Q328X	Y	Y
13-0043	*CFTR*	NM_000492	c.1418_1418delG	p.G473Efs*54	N	NA
13-0054	*CFTR*	NM_000492	**c.1520_1522delTCT**	**p.F508del**	Y	Y
14-3079	*CFTR*	NM_000492	c.3700A>G	p.I1234V	Y	Y
14-3072	*CFTR*	NM_000492	c.416A>T	p.H139L	Y	Y
14-3071	*CFTR*	NM_000492	c.3700A>G	p.I1234V	Y	Y
13-0090	*CDH23*	NM_001171930	^#^Missense		Y	Y
13-0102	*COL11A1*	NM_080630	c.2354G>A	p.G785E	Y	Y
13-0011	*DBT*	NM_001918	c.61_61delC	p.R21Afs*12	Y	Y
13-0046	*DBT*	NM_001918	c.773-2A>G	NA	Y	Y
13-0099	*DBT*	NM_001918	c.1195T>C	p.S399P	Y	Y
13-0104	*DBT*	NM_001918	c.1281+3A>G	NA	Y	Y
13-0107	*DBT*	NM_001918	c.137A>G	p.K46R	Y	Y
13-0127	*DBT*	NM_001918	c.773-2A>G	NA	Y	Y
13-0108	*DHCR7*	NM_001163817	c.861C>G	p.N287K	Y	Y
13-0100	*FGFR3*	NM_000142	**c.1138G>A**	**p.G380R**	Y	Y
13-0010	*GAA*	NM_000152	^#^Nonsense		Y	Y
13-0012	*GAA*	NM_000152	^#^Nonsense		Y	Y
13-0055	*GAA*	NM_000152	^#^Nonsense		Y	Y
13-0103	*GAA*	NM_000152	^#^Nonsense		Y	Y
13-0109	*GAA*	NM_000152	^#^Missense		Y	Y
13-0110	*GAA*	NM_000152	^#^Missense		Y	Y
13-0124	*GAA*	NM_000152	c.655G>A	p.G219R	Y	Y
13-0128	*GAA*	NM_000152	^#^Nonsense		Y	Y
13-0064	*GALT*	NM_000155	^#^Frameshift deletion		Y	Y
13-0125	*GALT*	NM_001258332	^#^Missense		Y	Y
13-0134	*GALT*	NM_001258332	^#^Missense		Y	Y
14-3078	*GALT*	NM_001258332	^#^Missense		Y	Y
14-3067	*GALT*	NM_001258332	^#^Missense		Y	Y
13-0101	*GBA*	NM_000157	c.152G>T	p.S51I	Y	Y
13-0130	*GBE1*	NM_000158	^#^Missense		Y	Y
13-0116	*GJB2*	NM_004004	**c.299T>C**	**p.W77R**	Y	Y
13-0116	*GJB2*	NM_004004	**c.506G>A**	**p.C169Y**	Y	Y
13-0111	*GLDC*	NM_000170	c.2113G>A	p.V705M	Y	Y
13-0060	*IDS*	NM_000202	^#^ Nonsense		Y	Y
13-0112	*MMAA*	NM_172250	^#^Nonsense		Y	Y
13-0126	*MMAA*	NM_172250	^#^Missense		Y	Y
13-0063	*MUT*	NM_000255	^#^Frameshift deletion		Y	Y
13-0105	*MUT*	NM_000255	c.329A>G	p.Y110C	Y	Y
13-0121	*MUT*	NM_000255	c.278G>A	p.R93H	Y	Y
14-3081	*MUT*	NM_000255	c.1160C>T	p.T387I	Y	Y
14-3080	*MUT*	NM_000255	**c.2200C>T**	**p.Q734X**	Y	Y
14-3070	*MUT*	NM_000255	c.2200C>T	p.Q734X	Y	Y
14-3065	*MUT*	NM_000255	c.1677-1G>C	NA	Y	Y
13-0044	*MYO7A*	NM_000260	c.5880-5882delCTT	p.F1961del	Y	Y
13-0117	*MYO7A*	NM_000260	c.2005C>T	p.R669X	Y	Y
13-0066	*OTC*	NM_000531	^#^ Missense		Y	Y
13-0057	*PAH*	NM_000277	^#^Missense		Y	Y
13-0113	*PAH*	NM_000277	^#^Nonsense		Y	Y
13-0114	*PAH*	NM_000277	^#^Missense		N	NA
13-0122	*PAH*	NM_000277	^#^Missense		Y	Y
13-0123	*PAH*	NM_000277	^#^Nonsense		Y	Y
13-0136	*PAH*	NM_000277	^#^Missense		Y	Y
14-3073	*PAH*	NM_000277	^#^Missense		Y	Y
13-0058	*POLG*	NM_001126131	c.2419C>T	p.R807C	Y	Y
13-0115	*PTPN11*	NM_002834	**c.188A>G**	**p.Y63C**	Y	Y
13-0026	*WAS*	NM_000377	c.91G>A	p.E31K	Y	Y
13-0027	*WAS*	NM_000377	c.100C>T	p.R34X	Y	Y

Mutations were either homozygous (regular), heterozygous (**bolded**) or hemizygous (underlined). #Exact mutation cannot be disclosed here being part of another ongoing unpublished study.

## Supplementary Materials

The following are available online at http://www.mdpi.com/2073-4425/9/5/267/s1, Table S1: IDP panel sequencing quality metrics; Table S2: Coverage per gene; Table S3: Amplicons with low overall performance; Table S4: List and details of undetected variants; Table S5: Examples of commercial NGS-panels.
