# Supplementary material for: Validation of Ion TorrentTM Inherited Disease Panel with the PGMTM Sequencing Platform for Rapid and Comprehensive Mutation Detection
Source: Genes (Basel). 2018 May 22;9(5):267. doi: 10.3390/genes9050267 (PMC5977207; doi:10.3390/genes9050267)
Supplement: Supplementary file 1 [file genes-09-00267-s001.pdf]

genes

Article

Validation of Ion Torrent™ Inherited Disease Panel with the PGM™ Sequencing Platform for Rapid and Comprehensive Mutation Detection

Abeer E. Mustafa [1,4], Tariq Faquih [2,4], Batoul Baz [1], Rana Kattan [4], Abdulelah Al-Issa [4], Asma I. Tahir [1], Faiqa Imtiaz [2], Khushnooda Ramzan [2], Moeenaldeen Al-Sayed [3], Mohammed Alowain [3], Zuhair Al-Hassnan [3], Hamad Al-Zaidan [3], Mohamed Abouelhoda [2,4], Bashayer R. Al-Mubarak [1,4,*] and Nada A. Al Tassan [1,4]

[1] Behavioral Genetics Unit, Department of Genetics, King Faisal Specialist Hospital & Research Center, P.O. Box 3354, Riyadh 11211, Saudi Arabia; almostafaa@sdl.com.sa (A.E.M.); bbaz@kfshrc.edu.sa (B.B.); bashairrashid@hotmail.com (A.I.T.); naltassan@kfshrc.edu.sa (N.A.A.T.)

[2] Department of Genetics, King Faisal Specialist Hospital & Research Centre. P.O. Box 3354, Riyadh 11211, Saudi Arabia; tfaquih@kfshrc.edu.sa (T.F.); fahmad@kfshrc.edu.sa (F.I.); kramzan@kfshrc.edu.sa (K.R.); mabouelhoda@yahoo.com (M.Ab.)

[3] Department of Medical Genetics, King Faisal Specialist Hospital & Research Centre, P.O. Box 3354, Riyadh 11211, Saudi Arabia; moeen@kfshrc.edu.sa (M.A.-S.); alowain@kfshrc.edu.sa (M.Al.); zhassnan@kfshrc.edu.sa (Z.A.-H.); hzaidan@kfshrc.edu.sa (H.A.-Z.)

[4] Saudi Human Genome Program, King Abdulaziz City for Science & Technology, Riyadh, Saudi Arabia; ra.kattan@gmail.com (R.K.); abdulbenissa@gmail.com (A.A.-I.)

* Correspondence: BAl-Mubarak@kfshrc.edu.sa

Table S1. IDP panel sequencing quality metrics.

Bases in Target Regions	Average Base Coverage Depth	Target Base Coverage at 1x	Target Base Coverage at 20x	Target Base Coverage at 50x
1509563	130.5	98.94%	95.46%	90.82%
1509563	182.5	99.14%	96.03%	93.48%
1509563	117.9	98.76%	94.01%	89.54%
1509563	136.59	98.07%	94.26%	89.93%
1509563	133.5	98.86%	94.67%	89.68%
1509563	146.5	98.91%	95.86%	92.11%
1509563	213.5	99.20%	96.89%	95.01%
1509563	179.2	98.99%	95.95%	93.10%
1509563	95.43	98.54%	92.14%	83.94%
1509563	101.1	98.66%	93.81%	86.36%
1509563	136.6	98.87%	94.31%	88.79%
1509563	172.7	98.68%	92.73%	88.32%
1509563	177.4	99.24%	96.65%	93.98%
1509563	198.6	99.23%	96.73%	94.28%
1509563	226.3	99.32%	97.12%	95.44%
1509563	25.73	98.16%	67.76%	68.13%
1509563	164.9	98.93%	95.69%	92.11%
1509563	138.4	98.87%	94.81%	89.69%
1509563	203	99.12%	96.78%	94.75%
1509563	67.53	98.63%	91.35%	79.25%
1509563	258.9	99.34%	97.04%	95.62%
1509563	91.93	98.94%	93.67%	84.47%
1509563	126.4	98.51%	92.97%	87.01%
1509563	282.7	99.41%	97.13%	95.93%
1509563	239.5	98.02%	77.80%	75.42%
1509563	251	99.30%	97.27%	96.13%
1509563	205.2	99.19%	96.55%	94.27%
1509563	276.3	98.89%	94.46%	89.56%
1509563	233.4	99.18%	96.13%	94.02%
1509563	177.4	99.16%	95.35%	91.73%
1509563	204.6	99.10%	96.05%	93.14%

1509563	249.5	99.29%	96.97%	95.62%
1509563	197.3	98.73%	92.58%	88.66%
1509563	132.3	98.17%	89.47%	83.44%
1509563	369.7	99.62%	97.36%	96.54%
1509563	123	98.94%	94.78%	88.56%
1509563	293.6	99.40%	97.08%	95.80%
1509563	213	99.25%	96.12%	93.00%
1509563	244.4	98.60%	93.48%	90.89%
1509563	268.7	98.78%	92.75%	90.49%
1509563	202.8	99.06%	96.37%	93.96%
1509563	233.4	99.25%	96.80%	95.16%
1509563	401.6	99.35%	97.35%	96.59%
1509563	124.4	98.86%	95.25%	89.75%
1509563	41.21	97.61%	73.12%	61.79%
1509563	81.14	98.46%	90.23%	80.39%
1509563	362.2	99.31%	96.98%	95.96%
1509563	120.7	98.82%	94.40%	88.21%
1509563	252.6	99.30%	96.80%	95.25%
1509563	166	99.13%	96.12%	93.17%
1509563	199.6	98.89%	95.04%	92.26%
1509563	137.5	98.80%	93.70%	88.54%
1509563	382.8	99.25%	96.28%	94.76%
1509563	176.8	99.08%	96.15%	93.24%
1509563	267.3	99.32%	96.70%	94.52%
1509563	273.6	99.32%	97.16%	95.57%
1509563	243	99.26%	97.15%	95.04%
1509563	183.5	99.07%	96.14%	92.67%
1509563	135.7	99.21%	96.34%	80.91%
1509563	45.57	98.30%	84.74%	85.47%
1509563	352.5	99.42%	97.28%	88.22%
1509563	183	98.64%	92.81%	91.88%
1509563	106.6	96.80%	76.43%	78.20%
1509563	378.8	99.15%	95.73%	92.73%
1509563	306.9	99.34%	97.05%	93.03%
1509563	225.2	99.08%	95.39%	93.64%
1509563	166.3	99.16%	95.03%	88.24%
1509563	223	99.30%	96.55%	81.78%
1509563	121	98.89%	93.47%	91.94%
1509563	42.15	97.71%	89.10%	85.09%
1509563	279.6	99.42%	96.78%	92.04%
1509563	167.3	99.19%	94.87%	89.31%
1509563	147.6	98.99%	95.17%	93.66%
1509563	141.3	98.94%	94.13%	90.87%
1509563	283.8	99.30%	96.77%	91.84%
1509563	179	99.20%	95.87%	91.43%
1509563	173.85	98.76%	92.89%	86.41%
1509563	204.9	98.94%	93.23%	81.54%
1509563	184.04	95.90%	90.14%	85.83%
1509563	140.3	97.43%	81.75%	78.25%
1509563	186.65	62.89%	93.25%	90.05%
1509563	175.5	98.44%	90.00%	84.49%
1509563	138.1	98.14%	92.13%	89.31%
1509563	205	98.40%	93.39%	86.29%
1509563	138.8	98.19%	89.80%	84.56%
1509563	248	98.72%	92.74%	90.33%
1509563	112.6	95.55%	91.04%	90.54%
1509563	180.8	95.51%	65.52%	68.04%
**1509563**	**191**	**98.36%**	**93.29%**	**89.34%**

Table S2. Coverage per gene.

Gene Name	Average Coverage(%)
ABCA4	99.34078031
ABCC9	97.99463602
ABCD1	99.04655172

ACADVL	99.21312261
ACTA2	93.7816092
ACTC1	99.60815047
ACTN2	96.82243361
ADA	99.74630542
AIPL1	99.58098224
AIRE	99.21896552
AKAP9	99.08301405
ALPL	99.5591616
AMT	99.60613027
ANK2	99.02724564
APC	99.35476856
APP	99.43915973
APTX	96.78376437
ARL6	99.48850575
ARSA	99.21264368
ASL	98.71264368
ASPA	99.46394984
ATL1	99.3136289
ATM	97.93413293
ATP2A2	99.57505071
ATP7A	98.76213934
ATP7B	99.5591133
ATXN1	99.07910751
ATXN2	96.45675972
ATXN7	97.25431034
BAG3	99.13464696
BCKDHA	99.25574713
BCKDHB	98.65747126
BEST1	99.38642583
BMPR1A	99.22504537
BTD	99.50957854
BTK	99.3584117
CA4	97.80747126
CACNA1C	103.5992544
CACNB2	98.41338259
CALR3	99.30946065
CAPN3	99.50076628
CASQ2	97.45320197
CAV3	99.38218391
CCDC39	99.56584565
CCDC40	99.5464191
CD113t-C	81.43103448
CDH23	98.79757344
CEP290	98.99676724
CERKL	98.54454023
CFTR	96.46952939
CHAT	94.93199234
CHD7	99.21209633
CHEK2	99.38052027
CHM	99.28735632
CHRNA1	99.56178161
CHRNB1	99.37088123
CHRND	99.41176471
CHRNE	98.40740741
CLCN1	96.35446793
CNGB1	98.58302764
COL11A1	98.42142111
COL11A2	96.52338214
COL1A1	95.5862069
COL1A2	98.26510938
COL2A1	99.42736009
COL3A1	99.50264057
COL4A1	99.30176703
COL4A5	99.33405172

COL5A1	96.37859195
COL5A2	99.49343186
COL7A1	99.54853129
COL9A1	99.0410509
CRB1	99.65648604
CRX	99.60775862
CTDP1	97.20197044
CTNS	99.40613027
CYP27A1	99.38074713
DBT	99.29939792
DCX	99.72605364
DES	98.6903313
DHCR7	99.07279693
DKC1	99.5154265
DLD	99.41431557
DMD	98.74857327
DNAH11	99.07557659
DNAH5	98.98606988
DNAH9	98.78019597
DNAI1	97.54549808
DNAI2	99.68481549
DNM2	99.13514455
DOK7	88.93678161
DSC2	99.34163474
DSG2	98.5545977
DSP	99.39926564
DYSF	99.12938403
ELN	99.46788371
EMD	98.90909091
ENG	96.76392573
EXT1	97.80096794
EYA1	97.11724138
EYS	98.96585865
F8	99.48347701
F9	99.51077586
FANCA	99.2535497
FANCC	99.44127936
FANCF	99.62579821
FANCG	99.57790549
FBN1	99.44926108
FBXO7	99.18874773
FGFR1	99.6743295
FGFR3	96.21609195
FMO3	99.71336207
FOXL2	83.87356322
FRG1	99.08908046
FRMD7	99.63278887
FSCN2	95.5348659
FXN	98.85057471
GAA	97.08416759
GALT	94.62260536
GATA4	97.85727969
GBA	99.60344828
GBE1	96.9306637
GCSH	99.63382594
GDF5	94.20977011
GJB2	99.85517241
GJB3	99.70689655
GJB6	99.53065134
GLA	98.70645447
GLDC	99.4216954
GNE	98.14896552
GNPTAB	98.76671891
GPC3	99.29643073
GPD1L	99.09814324

GPR143	95.61755486
GUCY2D	99.29118774
HBA2	92.00766284
HCN4	95.58422513
HEXA	99.52528736
HFE	99.62413793
HIBCH	99.04761905
HMBS	95.56168582
HR	96.43869732
IDS	99.2394636
IDUA	91.86206897
IKBKAP	99.52751284
IL2RG	99.46360153
IMPDH1	97.91354323
ITGB4	99.35123422
JAG1	98.33492976
JUP	98.82183908
KCNE1	99.77586207
KCNE2	99.59003831
KCNE3	99.70114943
KCNH2	94.18295019
KCNJ2	99.66666667
KCNQ1	97.47701149
KCNQ4	93.45977011
KIAA0196	98.54858934
KLHL7	98.19827586
KRAS	99.80114943
KRT14	99.81685824
KRT5	99.72485632
L1CAM	99.51577403
LAMB3	99.67784163
LAMP2	99.37988506
LDB3	96.70114943
LMNA	99.69020252
LRAT	99.49261084
LRRK2	98.563446
MAPT	93.31243469
MC1R	82.49753695
MECP2	98.07780725
MED12	98.99799307
MEN1	96.84815487
MERTK	97.8772578
MFN2	99.65217391
MLH1	97.95759017
MMAA	99.31877395
MMAB	98.71264368
MMACHC	98.72413793
MPZ	99.30268199
MSH2	99.17816092
MTM1	99.3862069
MUT	98.76657825
MYBPC3	99.51402299
MYH11	99.63385243
MYH6	98.69262782
MYH7	99.32081281
MYL2	99.65517241
MYL3	98.7164751
MYLK	99.17177522
MYO7A	98.99141393
MYOZ2	99.58748404
NF1	98.5408626
NF2	99.71206897
NIPBL	98.6032314
NKX2-5	98.14712644
NPC1	99.25788388

NPC2	99.12212644
NR2E3	97.68719212
NRAS	91.32183908
NSD1	99.65694076
OCA2	98.4453096
OCRL	97.35440613
OTC	99.10536398
PABPN1	83.46708464
PAFAH1B1	99.62422635
PAH	99.68199234
PAX3	99.34180278
PAX6	99.4448952
PCDH15	99.06134723
PEX1	96.87164751
PEX10	99.26666667
PEX13	99.4954023
PEX14	98.93545535
PEX19	99.64712644
PEX26	99.51954023
PEX3	99.2353297
PEX5	99.348659
PINK1	98.3256705
PKD1	98.06270915
PKHD1	98.77927443
PKP2	99.53639847
PLEC	97.41167109
PLN	99.46551724
PLOD1	99.08745627
PMM2	95.73180077
PMP22	99.33793103
POLG	99.53898726
PPT1	99.70019157
PRCD	97.96934866
PRKAG2	99.49952107
PROM1	98.79310345
PRPF31	98.84310345
PRPF8	99.58215962
PRPH2	99.73132184
PSEN1	99.66091954
PSEN2	96.36646383
PTCH1	99.39276703
PTPN11	98.24137931
RAF1	99.70833333
RAG1	99.64942529
RAG2	99.67126437
RAI1	99.17659352
RAPSN	98.00153257
RB1	98.47459165
RDH12	99.67471264
RET	99.11832319
RHO	91.51724138
ROR2	99.27936032
RP9	98.05363985
RPE65	99.27789047
RPGR	97.18304598
RPGRIP1	99.43087916
RPL11	99.39846743
RPL35A	98.90804598
RPS10	99.61685824
RPS19	95.14367816
RPS24	97.78787879
RPS26	99.73180077
RPS6KA3	99.432703
RPS7	97.6683087
RS1	99.64623244

RSPH4A	99.45916515
RSPH9	99.40689655
RYR1	97.11906186
RYR2	99.41177657
SALL4	97.48898467
SCN1B	89.55977011
SCN3B	95.37356322
SCN4B	99.67323481
SCN5A	99.63199234
SCN9A	99.11624027
SEMA4A	95.64477285
SERPINA1	99.61494253
SERPING1	99.62972085
SGCD	99.36015326
SH3BP2	98.107683
SIX1	97.49137931
SIX5	93.63218391
SLC25A13	96.88773946
SLC25A4	89.52745849
SLC26A4	99.60881226
SMAD3	96.79386973
SMAD4	99.78265413
SNCA	99.61685824
SNRNP200	99.57653713
SNTA1	93.78304598
SOD1	99.59961686
SOS1	97.2384494
SOX9	93.65163572
SPATA7	98.96751624
SPG7	99.18206897
STARD3	93.61460446
TAF1	98.81331748
TAZ	98.933687
TBX5	99.62474645
TCOF1	99.34874052
TGFBR1	97.84719405
TGFBR2	99.78951149
TMEM43	95.05977011
TNNC1	99.75533662
TNNI3	99.2112069
TNNT1	91.86453202
TNNT2	99.6653144
TNXB	96.64730135
TOPORS	99.50679206
TP53	99.5348659
TPM1	98.32123412
TRPP2	97.92944907
TSC1	99.4969574
TSC2	98.70729291
TTPA	98.90229885
TTR	99.71264368
TULP1	94.77701149
TWIST1	97.55172414
TXNDC3	99.07105538
TYR	98.2394636
USH1C	96.11912226
USH2A	99.37174638
VCL	97.91891892
VHL	93.20114943
WAS	95.21519796
WRN	98.73337102
WT1	91.23275862
**Average coverage**	**98.17483342**

Table S3. Amplicons with low overall performance.

Amplicon	Gene	Average_Cov	Median_Cov
AMPL333665053	*ABCC9*	0.40862069	0.19
AMPL332654447	*ACTN2*	0.221954023	0.1
AMPL332924504	*ANK2*	0.396781609	0.22
AMPL329137616	*ATM*	0.263448276	0.15
AMPL223533360	*ATM*	0.556206897	0.735
AMPL224357617	*ATM*	0.616091954	0.81
AMPL263006586	*ATXN2*	0.024827586	0
AMPL264529884	*ATXN7*	0.409885057	0.44
AMPL333434672	*CACNA1C*	0.352413793	0.11
AMPL332860700	*CACNA1C*	0.323333333	0.155
AMPL332795564	*CACNA1C*	0.603103448	0.725
AMPL316480986	*CD113t-C*	0.51045977	0.36
AMPL256851407	*CFTR*	0.412528736	0
AMPL263693016	*CFTR*	0.342298851	0
AMPL258800042	*CHAT*	0.155747126	0.05
AMPL259820389	*CLCN1*	0.21908046	0.13
AMPL268784639	*COL11A1*	0.084482759	0
AMPL267473214	*COL11A2*	0.100344828	0
AMPL261604288	*COL11A2*	0.477011494	0.26
AMPL226082590	*COL1A1*	0.062528736	0
AMPL253629491	*COL1A1*	0.443793103	0.25
AMPL266335107	*COL1A2*	0.444022989	0.24
AMPL267875911	*COL5A1*	0.178850575	0
AMPL264271893	*COL5A1*	0.462988506	0.17
AMPL266962722	*COL5A1*	0.520344828	0.555172414
AMPL269301385	*DNAH11*	0.494022989	0.155
AMPL262828421	*DNAH9*	0.38183908	0.125
AMPL263347778	*DNAI1*	0.509655172	0.56
AMPL264490187	*DOK7*	0.204252874	0.07
AMPL263906362	*DOK7*	0.145287356	0.11
AMPL264184718	*ENG*	0.273563218	0.29
AMPL317483006	*EYA1*	0.532758621	0.56
AMPL229282611	*FGFR3*	0.40183908	0.11
AMPL229752887	*FOXL2*	0.467241379	0.32
AMPL229746185	*FOXL2*	0.677586207	0.67
AMPL317490574	*GAA*	0.419425287	0
AMPL316565936	*GALT*	0.390114943	0
AMPL316923343	*GBE1*	0.293103448	0.21
AMPL320478284	*GDF5*	0.367701149	0.145
AMPL332489562	*HCN4*	0.392183908	0.245
AMPL315970732	*HMBS*	0.436321839	0.285
AMPL317831668	*HR*	0.680344828	0.75
AMPL328551274	*IDUA*	0.011494253	0
AMPL316597329	*IMPDH1*	0.685977011	0.84
AMPL316229103	*JAG1*	0.530689655	0.64
AMPL312881912	*KCNH2*	0.231494253	0.115
AMPL312732660	*KCNH2*	0.260229885	0.285
AMPL314511384	*KCNQ4*	0.227011494	0.05
AMPL313253246	*KIAA0196*	0.508965517	0.45
AMPL313342676	*LDB3*	0.695747126	0.697873563
AMPL313342915	*LDB3*	0.766896552	0.79
AMPL313402560	*MAPT*	0.198965517	0
AMPL313034049	*MC1R*	0.344827586	0.205
AMPL314723961	*MC1R*	0.431034483	0.3
AMPL314594164	*MED12*	0.697356322	0.708678161
AMPL231208212	*MEN1*	0.580114943	0.545
AMPL313004422	*MERTK*	0.581264368	0.815
AMPL233218426	*MLH1*	0.498505747	0.47
AMPL313769556	*MYO7A*	0.528045977	0.54
AMPL238333700	*NF1*	0.513678161	0.52183908
AMPL292477743	*NIPBL*	0.144712644	0.11
AMPL257613650	*NRAS*	0.502298851	0.255
AMPL320308769	*OCRL*	0.357471264	0

AMPL320109858	*PABPN1*	0.057471264	0.02
AMPL320132837	*PABPN1*	0.161609195	0.135
AMPL318283880	*PEX1*	0.358045977	0
AMPL321180668	*PEX1*	0.526666667	0.39
AMPL319536438	*PKD1*	0.532988506	0.491494253
AMPL319473951	*PKHD1*	0.233563218	0.25
AMPL318323320	*PLEC*	0.235977011	0.21
AMPL320499907	*PLEC*	0.674367816	0.685
AMPL320625284	*PLEC*	0.714942529	0.815
AMPL323884687	*PSEN2*	0.435172414	0.405
AMPL326400261	*RHO*	0.189770115	0.12
AMPL324248910	*RPGR*	0.084942529	0.09
AMPL326287708	*RYR1*	0.053678161	0
AMPL326287558	*RYR1*	0.339655172	0.13
AMPL324726906	*RYR1*	0.363908046	0.35
AMPL323496723	*RYR1*	0.522643678	0.54
AMPL325586375	*SALL4*	0.505977011	0.155
AMPL332516827	*SCN1B*	0.347816092	0
AMPL323504700	*SEMA4A*	0.162758621	0.12
AMPL323672232	*SIX5*	0.347931034	0.2
AMPL323733372	*SLC25A13*	0.487471264	0.34
AMPL324304978	*SLC25A4*	0.14862069	0.13
AMPL272494146	*SMAD3*	0.680804598	0.725
AMPL327047268	*SOS1*	0.315402299	0
AMPL328121251	*SOS1*	0.535402299	0.33
AMPL327622945	*SOX9*	0.617701149	0.64
AMPL327622743	*SOX9*	0.634367816	0.655
AMPL333358745	*STARD3*	0.349655172	0
AMPL303260558	*TAF1*	0.619885057	0.695
AMPL332264093	*TMEM43*	0.347356322	0
AMPL326487987	*TNNT1*	0.094827586	0
AMPL331572195	*TNXB*	0.457931034	0.13
AMPL331905893	*TNXB*	0.643678161	0.51
AMPL331814080	*TNXB*	0.693563218	0.595
AMPL331947764	*TNXB*	0.666896552	0.64
AMPL331756060	*TNXB*	0.760229885	0.84
AMPL327801779	*TULP1*	0.092528736	0
AMPL327024814	*USH1C*	0.051954023	0
AMPL328293814	*USH2A*	0.622528736	0.72
AMPL326701550	*VCL*	0.372528736	0.236264368
AMPL262401836	*WAS*	0.320229885	0.225
AMPL266347323	*WT1*	0.195287356	0.22

Table S4. List and details of undetected variants.

Case-ID	Gene	Transcript-ID	chromosome	Start	End	c.DNA	Protein	Context Sequence	Site Coverage (%)	HOMOPOLYMER (Y/N)	High-GC Content (Y/N)	Reason for Variant Detection Failure
SGP2013-0133	*ASPA*	NM_000049	17	3397706	3397706	#Frameshift insertion		AAAGTTGATTACCCCCGG GATGAAAATGGAG	46x	Y	N	presence in homoploymer region
SGP2014-3064	*ASPA*	NM_000049	17	3384972	3384973	#Frameshift insertion		ATTTG GTCCAAAAGACAGTGAA GATT CCTAT	36x	Y	N	presence in homoploymer region
SGP2013-0091	*BCKDHB*	NM_000056	6	80838889	80838892	c.286_288delGAA		TCAGTAATATTTGGTGAA GATGTTGCCTTTG	9x	N	N	poor coverage
SGP2013-0092	*BCKDHB*	NM_000056	6	80816411	80816411	c.1A>T	p.M1L	CGGTGGTGAGCGGGGAT GGCGGTTGTAGCGG	2x	Y	Y	poor coverage, presence in homoploymer and GC-rich region
SGP2013-0043	*CFTR*	NM_000492	7	117199543	117199543	c.1418_1418delG	p.G473Efs*54	TAATGGTGATTATGGGAG AACTGGAGCCTTC	3x	Y	N	poor coverage & presence in homoploymer region
SGP2013-0114	*PAH*	NM_000277	12	103260390	103260390	#Missense		GGCGGTAGTTGTAGGCA ATGTCAGCAAACTG	16x	N	N	poor coverage

Table S5. Examples of commercial NGS-panels.

Test Provider	Type of test	Platform	Link
Illumina	Group of disorders (552 genes)	Illumina	https://www.illumina.com/products/by-type/clinical-research-products/trusight-inherited-disease.html
Fulgent Diagnostics	Individual disorder	Illumina HiSeq 4000	https://www.fulgentgenetics.com/testgroup/panel
Center for Genomics and Transcriptomics	Group of disorders (634 genes)	in-Solution Agilent technology (SureSelectXT)	http://www.cegat.de/en/diagnostics/diagnostic-panels/epilepsy-metabolic-and-brain-development-disorders/#tab-id-2
Blueprint Genetics	Group of disorders (354 genes)	OS-Seq™	http://metabolic-disorders.blueprintgenetics.com/panels/comprehensive-metabolism-panel/
Otogenetics	Group of disorders (4500 genes)	Illumina HiSeq	https://www.otogenetics.com/genepanels/inherited-disease-panel

© 2018 by the authors; licensee MDPI, Basel, Switzerland. This article is an open access article distributed under the terms and conditions of the Creative Commons Attribution (CC BY) license (http://creativecommons.org/licenses/by/4.0/).
